# Biometric Security: A Novel Ear Recognition Approach Using a 3D Morphable Ear Model

**DOI:** 10.3390/s22228988

**Published:** 2022-11-20

**Authors:** Md Mursalin, Mohiuddin Ahmed, Paul Haskell-Dowland

**Affiliations:** School of Science, Edith Cowan University, Perth 6027, Australia

**Keywords:** ear biometrics, detection, 3D morphable model, recognition

## Abstract

Biometrics is a critical component of cybersecurity that identifies persons by verifying their behavioral and physical traits. In biometric-based authentication, each individual can be correctly recognized based on their intrinsic behavioral or physical features, such as face, fingerprint, iris, and ears. This work proposes a novel approach for human identification using 3D ear images. Usually, in conventional methods, the probe image is registered with each gallery image using computational heavy registration algorithms, making it practically infeasible due to the time-consuming recognition process. Therefore, this work proposes a recognition pipeline that reduces the one-to-one registration between probe and gallery. First, a deep learning-based algorithm is used for ear detection in 3D side face images. Second, a statistical ear model known as a 3D morphable ear model (3DMEM), was constructed to use as a feature extractor from the detected ear images. Finally, a novel recognition algorithm named you morph once (YMO) is proposed for human recognition that reduces the computational time by eliminating one-to-one registration between probe and gallery, which only calculates the distance between the parameters stored in the gallery and the probe. The experimental results show the significance of the proposed method for a real-time application.

## 1. Introduction

**Figure 1:**
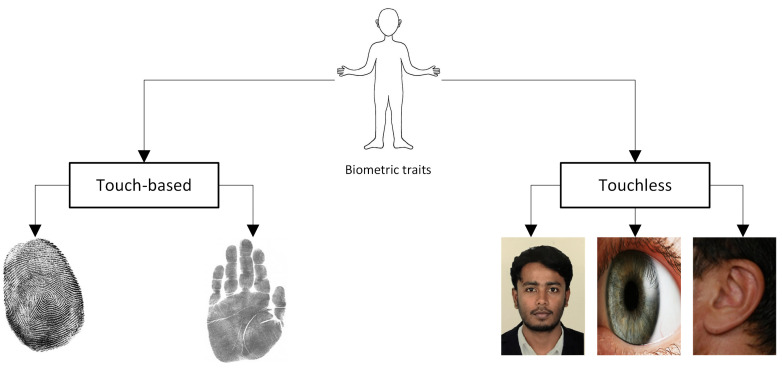
Various biometric traits for human recognition.

Biometrics is a critical component of cybersecurity that identifies persons by verifying their behavioral and physical traits. It is the most precise and powerful physical security solution for identity verification presently in use. In biometric-based authentication, each individual can be correctly recognized based on their intrinsic behavioral or physical features. Biometrics systems based on physiological attributes such as the face [1], fingerprint [2], iris [3], palm prints [4], and ear [5] have been described by researchers [6]. Examples of these physiological biometrics qualities are shown in Figure 1. If someone tries to get into the biometric security system, it scans them, analyses their traits, and compares them to previously recorded information. The individual is given access to the facility or equipment when there is a match found.

**Figure 2:**
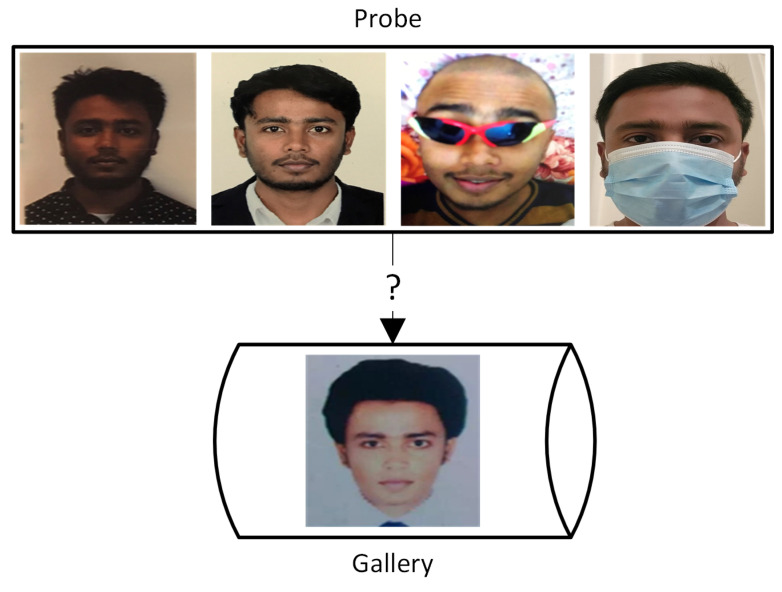
Face recognition challenges. All of these images (same person) are taken within 2–3 years.

Biometrics can be categorized into touch-based and touchless. The fingerprint is one of the most popular touch-based biometrics used presently. However, with the advancement of technology, hackers can mimic fingerprints and access important information. Moreover, the weather conditions such as rain, snow, or humidity can cause problems with the fingerprint-based security system. Furthermore, the quality of the hardware degrades over time. Apart from touch-based, facial recognition is considered the prominent touchless biometrics. Although it shows advantages over fingerprints, it faces numerous challenges, including illumination, and poses variations, ages, etc. A sample challenge in the facial recognition system is illustrated in Figure 2, where the images (probe) of the same person show lots of deviation from the original stored image (gallery). Therefore, the face-based approach has to be robust against those situations which infer the need for additional biometric traits. The human ear is considered a crucial biometric, showing highly distinguishing features. Researchers found that even identical twins have different ear shapes [7]. The advantage of ear image analysis over other biometric traits, such as faces, iris, fingerprints, and palm prints can be attributed to its ease of capture, invariance to expressions, and stability over time [8]. Due to these benefits, ear images can be used for various purposes such as biometric identification, clinical asymmetry research, genetic connection investigation, and gender recognition [9,10,11,12].

Generally, touch-based biometrics such as fingerprints and palm prints should be avoided for public safety due to the rising concern about the COVID-19 pandemic. As a result, a touchless biometric system for person recognition in real-world applications such as office attendance, access management, banking, and surveillance is in high demand. Faces are likewise non-intrusive biometrics but have a barrier because they are often hidden behind masks. Due to its non-intrusive nature, the ear is a valuable biometric in this situation where a facial mask covers the face, but the ear region is visible.

Although 2D ear image analysis is more widespread because of its ease of computation, it has certain limitations, such as sensitivity to illumination and poses variations. Moreover, the shape information is limited for a small object, such as an ear. On the contrary, a 3D image provides much more shape information, even for a small object. Therefore, 3D ear image analysis can be a promising prospect for human recognition.

In an ear-based recognition system, a gallery is referred to as a set of images indicating all individuals known to the system. This work provides one image per person to create the gallery. The images of the same individual not in the gallery are called probe images. In this work, we evaluated two recognition tasks. The first task is identification, where the system notices which person from the gallery is revealed on the probe image. The second task is verification, where a person declares to be a certain gallery image. The system determines if the probe and the gallery image show the same person.

Usually, in the literature, each probe is used to register using the registration algorithm for all the gallery images. Therefore, it is very time-consuming and computationally expensive. This work develops an algorithm named YMO that needs only one registration to deform the 3DMEM towards a given query image and calculate the shape parameters. After computing the parameters, this approach only calculates the distance between the parameters stored in the gallery and the probe without requiring further registration steps. The recognition result is verified based on a threshold. The contribution of this work can be summarised as follows,

- A novel recognition algorithm named YMO is developed to match the probe and gallery, reducing computational time.
- The performance of different distance metrics for ear recognition is demonstrated.
- A comparative study shows the comparable performance of our 3D ear recognition method.

## 2. Literature Review

The existing 3D ear recognition method mostly used either local features or global features for representing the feature space. These features were extracted after pairwise registering the ears. As a result, the recognition process becomes computationally heavy when a given probe image needs to register with all the gallery images. Before 2005, existing ear recognition methods did not show any performance prediction, either theoretically or experimentally. The existing 3D ear recognition method can be categorized based on feature types such as local, global, and fusion of local and global features. This section briefly explains the different existing methods that use these features.

### 2.1. Local Feature

Chen et al. [13] first proposed a new representation of a 3D ear based on an integrated local feature to recognize human ears. They defined the features using local minimum and maximum shape indexes from principal curvatures. An initial correspondence of the local surface patches was established by comparing local surface patches between an offline model and test images. The performance of their method was evaluated on 52 subjects using the cumulative match characteristic (CMC) curve. Later, they [14,15] introduced a method for ear recognition by combining feature embedding and support vector machine (SVM). The local surface patch representation established the correspondence between the model and test images. To reduce the high dimensionality of the feature vector, they applied a feature embedding algorithm. The similarity between a model and test pair was calculated by searching the nearest neighbors from the low dimensional embedded features. The similarities for all model and test pairs were ranked using the SVM algorithm that generates a shortlist of candidate models for verification.

To encode 2D and 3D local features for ear recognition, Chen et al. [16] introduced the texture and depth scale-invariant feature 100 transform (TDSIFT). The TDSIFT showed its superiority over the conventional scale-invariant feature transform (SIFT) descriptor by fusing 2D and 3D local information. They applied a key-point detection method on 2D images and then projected the corresponding key point on the co-registered 3D images to form the TDSIFT descriptor.

By identifying key points that utilize the curvilinear structure in 2D ear pictures projected to the co-registered 3D ear images, Ganapathi et al. [17] developed a local feature description-based technique for human recognition. A feature descriptor vector was calculated from the neighborhood around each mapped key point in 3D. The correspondence between each pair of probe and gallery images was established by using the Iterative Closest Point (ICP) algorithm [18]. The registration score was used to determine the matching score. Later they [19] proposed a feature extraction method using geometric statistics that eliminates the dependency on 2D co-registered images. However, to enhance the feature extraction, the authors [20] extracted additional descriptors by utilizing neural network-based auto-encoders and local statistics of the depth images. In addition, the authors [18] proposed a multi-modal approach that used 2D images to identify key points and co-registered 3D images to extract features from the key points. They analyzed six key-point detectors with the ICP algorithm and reported the performance.

A 3D local feature-based (L3DF) method that merged the ear and face for human recognition was proposed by Islam et al. [21]. They detected key points based on asymmetrical variations in depth from both faces and ears. An altered ICP was applied to align the probe and gallery dataset. They utilized the weighted sum during the fusion to weigh more on face futures over-ear data. The recognition performance for their work was mostly dependent on the face. Therefore, the authors [22] proposed a method for 3D ear recognition that did not require facial features. They applied an AdaBoost algorithm to detect ears from 2D profile face images that were projected to the co-registered 3D images to crop the 3D ear. A speeded-up robust feature (SURF) feature-based method was proposed by Prakash et al. [23]. The SURF features were extracted from the co-registered 3D images.

To extract local features directly on 3D ear point clouds, Sun et al. [24] proposed a method using a Gaussian-weighted average of the mean curvature of each point. They presented an optimal selection of the salient key points utilizing the Poisson disk sampling. Subsequently, the authors created a local feature descriptor of each salient key point by fitting a surface to the neighborhood of each salient key point using the quadratic principal manifold method. Later, the authors [25] investigated the local shape feature combined into the joint α-entropy of the minimum spanning tree (MST). They used their previous technique to detect salient points from the 3D ear image and fit the neighborhood of each salient point to a single-value quadric surface. Each salient point’s local shape feature vector was specified as the sampling depth set on the parametric node of the quadric surface. They constructed the MST on the matched key points for every pair of gallery ears and probe ears. Eventually, the authors reduced the total edge weight of MST to determine the similar pair if its joint α-entropy value is small.

A method using shape information to arrange the 3D ear data in a hierarchical categorization was presented by Maity et al. [26], where the ear was segmented by applying an active contour algorithm with a tree-structured graph. These 3D ears were partitioned into different categories based on geometric shapes, including round, oval, rectangular, etc. They used indexing methods with balanced split (KD tree) and unbalanced split (pyramid tree) data structures to distinctly categorize the database. Zeng et al. [27] presented a similar shape-based method. They created the 3D Center-Symmetric Local Binary Patterns (CS-LBP) features and utilized a coarse-to-fine approach for 3D salient point matching. The matching scores were computed using the average Earth mover’s distance (EMD) distances for 3D ear recognition. Later the authors [28] improved the method by using a modified iterative closet point (MICP). The authors computed three descriptors: LBP descriptor, 3D LBP descriptor, and 3D CS-LBP descriptor for feature extraction and matching. For leading additional local feature information into global registration, Zhang et al. [29] used a one-step ICP local surface variation (LSV) algorithm for a 3D ear matching scheme. They applied data normalization to eliminate the background noise.

### 2.2. Global Feature

By generating 3D data from video frames, Cadavid et al. [30] suggested a technique for ear recognition. For 3D ear identification systems, they used two algorithms called shape from shading (SFS) and structure from motion (SFM). The ear region was segmented from each frame in a video sequence using interpolation of ridges and ravines. They reconstructed 3D shapes by tracking key points across video frames and using the factorization method. This reconstructed 3D ear model was matched using ICP for recognition. Later they [31] increased the number of subjects and applied the SFS method for recognition. However, they did not perform any robustness again poses or occlusions. A similar video-based approach was proposed by Mahoor et al. [32] for multi-modal face and ear recognition. They reconstruct 3D images from a series of video frames using the SFS method. The 3D models were registered using the ICP algorithm. The Active Shape Model was used to derive a collection of facial landmarks from frontal facial photos for 2D face recognition. Then, at the positions of face landmarks, the response of facial images to a sequence of Gabor filters is determined. The best match was determined by comparing the Gabor features of a probe face image to those of the reference models. The ear recognition and face recognition modality match scores are combined to improve the system’s overall recognition rate.

A 3D ear recognition technique was presented by Passalis et al. [33] utilizing a general annotated ear model (AEM). This AEM was registered and placed into each ear to generate a biometric signature with 3D data storage. After the registration process, depth and normal images were generated and concatenated to form the biometric signature coefficients. The $L^{1}$ metric was used to compare the coefficients between two images. For further improvement, the authors [34] proposed a unified method that fuses 3D facial and ear data. They used an annotated deformable model fitting to the data and calculated wavelet coefficients from the geometry image for the biometric signature.

A theoretical approach for calculating the similarity of equivalent feature sets was presented by Tre et al. [35]. Their research aimed to demonstrate how computational intelligence could improve ear recognition. They proposed bipolar data modeling and aggregation methods to represent the data to improve the performance against noises. The similarity between the two ear images was measured by calculating the Minkowski distance and managing the hesitation caused by poor image quality [36]. They also proposed a hierarchically structured comparison method for features.

## 3. Proposed Method

**Figure 3:**
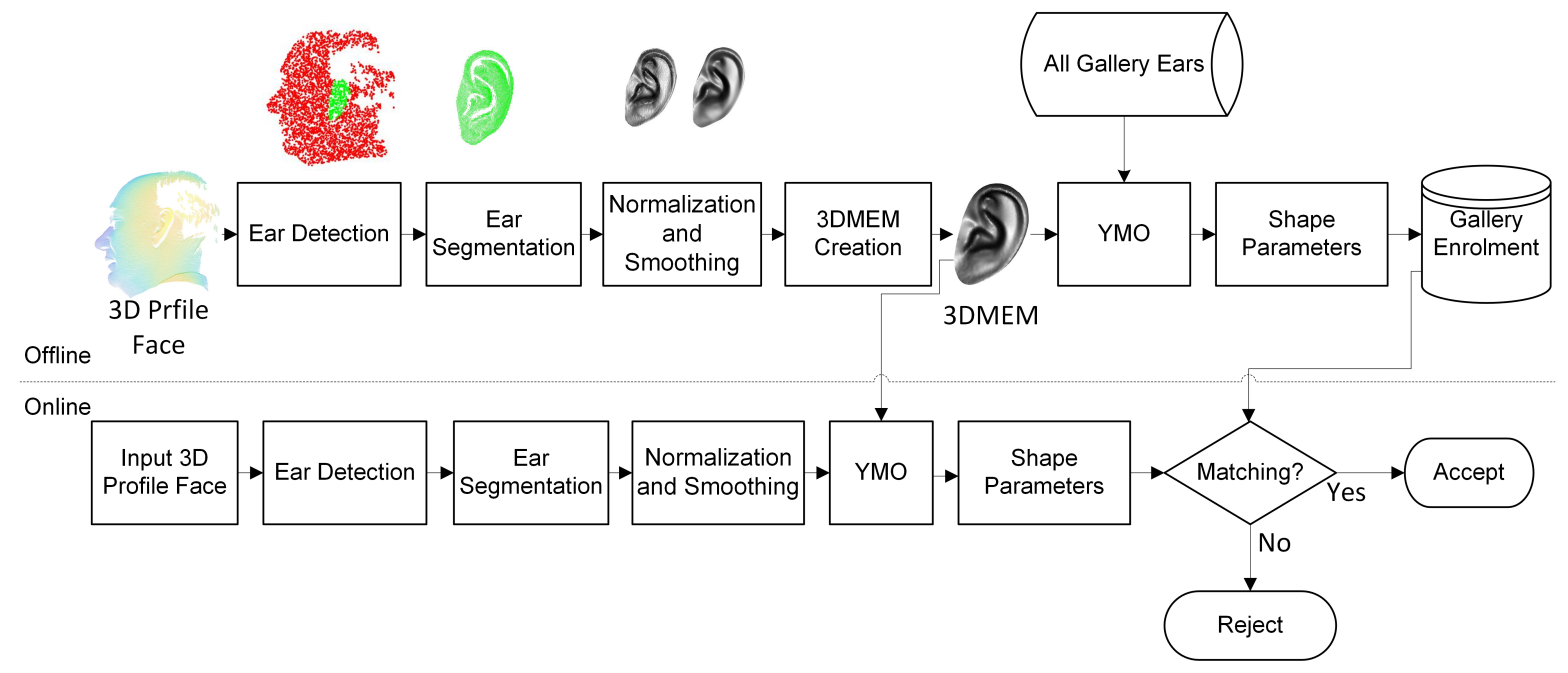
Block diagram of the proposed ear recognition method.

This work proposed a complete pipeline for ear recognition from 3D profile images. The profile is considered a left-side 3D image in this work. The first step is to detect and extract the ear from the profile face image. This work used a deep learning-based method for ear detection that can detect ears from the 3D point cloud representation of the profile face. The next step is to create a 3DMEM from the extracted 3D ear images. A combination of rigid ICP and a variant of a non-rigid ICP algorithm are used to register two ear images in a way so that each point of one ear should correspond to the other ear. This representation is known as dense correspondence. After achieving dense correspondence, we can use linear representation to apply statistical analysis, which facilitates the creation of a statistical shape model known as the 3D morphable ear model (3DMEM). This 3DMEM can be used as a generic representation of the ear shape and can be used for parameterization for a new instance. Next, a novel matching technique is proposed to extract the shape parameters. This work demonstrates various distance metrics to find the best solution. The recognition is considered successful when the distance value satisfies the threshold condition. The proposed ear recognition method is illustrated in Figure 3.

### 3.1. Dataset Preparation

The proposed technique was validated on the University of Notre Dame (UND) J2 database. This database is considered one of the most extensively accessible ear databases, with 1800 samples from 415 subjects. The images in the database are affected by pose changes, scaling, and occlusions due to earrings and hair. The recognition system requires at least one gallery image per person. In this work, we only considered subjects with two or more samples, so the gallery should contain one image per subject. Among 415 subjects of the UND J2 dataset, we found 404 subjects that satisfied our condition. For ear recognition, the first task is to create a gallery and probe dataset from the chosen 404 subjects with 1780 samples. A gallery dataset consists of 404 arbitrarily chosen images from each subject, and the probe comprises the remaining 1376 images.

### 3.2. Ear Detection

The ear detection from 3D profile faces is performed by adopting our previously developed fully automated deep learning-based algorithm named EarNet [37]. The EarNet is a modified version of the PointNet++ [38] architecture. EarNet is lighter than PointNet++, making the computation significantly faster. Additionally, a data augmentation block was included to rotate the full 3D ear point cloud. This rotation was performed with respect to the x and y axes. This augmentation leverage the understanding of a given profile face object and enhances the performance of ear detection in 3D point clouds. The EarNet is trained from scratch utilizing 20,000 synthetic data (training and testing split was 80% and 20%). We empirically selected the hyperparameters. The experimental observation showed the optimal batch size was 16. We assigned the number of data points of each scan as 4096. The optimizer was set as Adam [39] with a momentum of 0.9. We choose the initial learning rate as $10^{-3}$. To improve the robustness, we used transfer learning to the network utilizing 150 real 3D scans (arbitrarily selected from the UND J2 dataset). We applied rotation augmentation during transfer learning. All experiments were conducted in the Lambda Blade machine with GPU 8× 1080 Ti GeForce GTX 1080 Ti. The EarNet shows 100% detection accuracy in the UND J2 dataset. More details can be found in [37,40].

### 3.3. Morphable Model Generation

After extracting the ear from a 3D profile face image, each ear image is parameterized so we can apply statistical operations [41]. This parameterization is achieved by morphing a template to every ear in the database. This is an iterative process where the template is deformed using a non-rigid ICP algorithm [42]. To select a template, we first randomly chose one sample ear from the ear database and registered it with all the remaining ears. After registering, we calculate the mean ear. This mean ear is again used as a new template, and we continue the same procedure again by registering all the ear image with the new template. So the output of the registration step is the set of reparameterized ear images where each point of the ear semantically corresponds with the other ears. Therefore, we can apply linear operations to these registered images. Now it is possible to create a 3DMEM using these densely corresponded ears. We reduce the dimensionality by using principal component analysis to construct the 3DMEM. By varying the shape parameters of the 3DMEM, we can generate novel ear-shaped instances. Let, the matrix of all densely corresponded ears $\Gamma =[v_{1},v_{2},\ldots ,v_{N}]$, where $v={\left[x_{1},\ldots ,x_{n},y_{1}\ldots ,y_{n},z_{1},\ldots z_{n}\right]}^{T}$ and number of vertices n = 1,…, N. Now, the statistical shape model (*M*) can be expressed as,

(1) $$M_{i}=\mu +\sum_{i=1}^{d}\alpha_{i}U_{i}=\mu +\alpha U$$

We can calculate the mean shape (μ) from the densely corresponded ears using the following equation,

(2) $$\mu =\sum_{i=1}^{N}e_{i}$$

The row-normalized matrix *R* is computed by subtracting the μ from each 3D ear image.

(3) R=Γ−μ

The eigenvalue is computed by applying a singular value decomposition method named svd. Here, *U* stands for principal components, *S* represents the diagonal matrix of eigenvalues, and *V* implies the corresponding loading.

(4) [U,S,V]=svd(R)

where α represents the shape parameters, which are applied to alter the shape, and *d* is the number of principal components. The shape parameter α is computed utilizing the following equation,

(5) $$\alpha =U^{T}\left(m-\mu \right)$$

### 3.4. You Morph Once Algorithm

The YMO algorithm starts with a rigid registration process followed by shape deformation. In this work, we adopted a fitting algorithm proposed by [43] for face deformation. However, we updated the method with different distance calculations for ear model deformation. The purpose is to deform the mean ear shape from the 3DMEM to minimize the distance between the query ear QE and the deformed mean ear EM. The query ear after vectorization is parametrized by the statistical model as $qm_{i}=U\alpha_{i}+\mu Y$, where the vector $\alpha_{i}$ consists of parameters. These parameters can vary the model’s shape in the *i*th iteration. The $\alpha_{i}$ is set to zero, and the mean ear of the 3DEM characterizes the deformable model $EM_{i}$ in the initialization step. Each iteration starts with a registration step where the input ear QE is registered to the model $EM_{i}$. In this step, an approximate correspondence between the $EM_{i}$ and the QE is computed with a rigid transformation. The correspondence is determined by analyzing the Nearest Neighbor (NN) of each point of $EM_{i}$ in QE following a k-d tree data structure. Suppose *d* represents the Spearman distance (see section Distance metrics) between the corresponding query ear and the model. The outliers are defined as points on $QE^{\prime }$ whose NN distance with $EM_{i}$ is more significant than a threshold th where $th=\bar{d}+2\sigma d$ and remove them from registration. This measure confirms that outliers do not influence the registration process. Next, the QE is translated to the μ of the model and is rotated to align with $EM_{i}$. Here, QEr denotes the corresponding and registered query ear. In the following step, the model $EM_{i}$ is deformed to fit the registered query ear QEr as

(6) $$\alpha_{i}=min_{\alpha_{i}}||Me-qe_{r}{\left|\right|}_{2}+\lambda ||\alpha_{i}-\alpha_{i-1}{\left|\right|}_{2}$$

where $Me=U*\alpha_{i}+\mu \gamma$, is calculated from the morphable ear model. The iterative process is completed when the residual error (η) between $qm_{i}$ and $qe_{r}$ is less than equal to $10^{-5}$. A stiffness weight λ was used to regularize the shape. In this experiment the λ was set to 0.6. After deforming the 3DMEM to the probe, the last step is to calculate the distance between the deformed 3DMEM with all of the gallery shape parameters. In this work, the Spearman distance is used to find the distance. The minimum distance is considered as the matching pair between the probe and the gallery. The pseudocode is shown in Algorithm 1.

**Algorithm 1** YMO Algorithm**Require:**3DMEM, QE, GAL**Ensure:**QE is upward facing  $EM_{0}=\mu$  i=1  **while** $e_{i}>\eta$
**do**    $QE^{\prime }=dist_{nn}\left(QE,EM_{i}\right)<th$    $QEr=rigidICP(QE^{\prime },EM_{i})$    $Me=U*\alpha_{i}+\mu \gamma$    $\alpha_{i}=min_{\alpha_{i}}||Me-qe_{r}{\left|\right|}_{2}+\lambda ||\alpha_{i}-\alpha_{i-1}{\left|\right|}_{2}$    $qm_{i}=U\alpha_{i}+\mu Y$    $e_{i}=dist_{sp}\left(m^{i}-q_{r}\right)$    i++  **end while**  Calculate the SP distance between DEM and GAl  Find the min(SP)  **if** min(SP) < thVal **then**    Match found in the gallery.  **else**    Not matched!  **end if**

### 3.5. Gallery Enrollment

The first task for any recognition is to create a gallery database of each individual to match the probe image. This work uses a novel approach to create the gallery database where a fitting algorithm is utilized to deform the 3DMEM as close to a given individual ear image. After the deformation of the 3DMEM, the parameters are calculated and stored in the gallery database. These parameters are used to transform the 3DMEM toward the given input image.

### 3.6. Evaluation Metrics

#### 3.6.1. Distance Metrics

A distance metric is a function that shows how far apart two observations are from one another. This study employs a number of different distance measures, including the Euclidean and normalized Euclidean distances, as well as the Mahalanobis, Minkowski, city block, Chebyshev, cosine, correlation, Spearman, and Jaccard distances. Given a data matrix *X* that is represented by m(1×n) row vectors $x_{1},x_{2},\ldots ,x_{m}$. As well as a m×n data matrix *Y*, which is represented as m(1×n) row vectors $y_{1},y_{2},\ldots ,y_{m}$. The following definitions describe the distances between the vectors $x_{s}$ and $y_{t}$,

Euclidean distance (E):

(7) $$E=\sqrt{\left(x_{s}-y_{t}\right){\left(x_{s}-y_{t}\right)}^{\prime }}$$

Standardized Euclidean distance (SE):

Here, *V* is the n×n diagonal matrix where ${\left(S\left(j\right)\right)}^{2}$ is the *j*th diagonal element, where for each dimension, *S* represents a vector of scaling factors.

(8) $$SE=\left(x_{s}-y_{t}\right)V^{-1}{\left(x_{s}-y_{t}\right)}^{\prime }$$

Mahalanobis distance (MH):

(9) $$MH=\left(x_{s}-y_{t}\right)C^{-1}{\left(x_{s}-y_{t}\right)}^{\prime }$$

where C represents the covariance matrix.

Minkowski distance (MN):

(10) $$MN=\sqrt[{p}]{\sum_{j=1}^{n}{\left|x_{sj}-y_{tj}\right|}^{p}}$$

City block distance (CT):

(11) $$CT=\sum_{j=1}^{n}\left|x_{sj}-y_{tj}\right|$$

Chebychev distance (CH):

(12) $$CH=max_{j}|x_{sj}-y_{tj}|$$

Cosine distance (CS):

(13) $$CS=1-\frac{x_{s}y_{t}^{\prime }}{\sqrt{\left(x_{s}x_{s}^{\prime }\right)\left(y_{t}y_{t}^{\prime }\right)}}$$

Correlation distance (CR):

(14) $$CR=1-\frac{\left(x_{s}-{\bar{x}}_{s}\right){\left(y_{t}-{\bar{y}}_{t}\right)}^{\prime }}{\sqrt{\left(x_{s}-{\bar{x}}_{s}\right){\left(x_{s}-{\bar{x}}_{s}\right)}^{\prime }}\sqrt{\left(y_{t}-{\bar{y}}_{t}\right){\left(y_{t}-{\bar{y}}_{t}\right)}^{\prime }}}$$

Spearman distance (SP):

(15) $$SP=1-\frac{\left(\xi_{s}-{\bar{\xi }}_{s}\right){\left(\xi_{t}-{\bar{\xi }}_{t}\right)}^{\prime }}{\sqrt{\xi_{s}-{\bar{\xi }}_{s}){\left(\xi_{s}-{\bar{\xi }}_{s}\right)}^{\prime }}\sqrt{\left(\xi_{t}-{\bar{\xi }}_{t}\right){\left(\xi_{t}-{\bar{\xi }}_{t}\right)}^{\prime }}}$$

where $\xi_{s}$ and $\xi_{t}$ represent the coordinate-wise rank vectors of $x_{s}$ and $x_{t}$.

#### 3.6.2. Identification Metrics

For ear recognition, the shape is represented by a set of coefficients, aka shape parameters $\alpha ={\left(\alpha_{1},...,\alpha_{n}\right)}^{T}$. Several distance metrics are used to evaluate the comparison between the gallery and probe ears $e_{1}$ and $e_{2}$.

The performance of the proposed approach is evaluated using the identification rate, and verification accuracy, where verification accuracy is defined as follows:

(16) $$Verification\;Accuracy=(100-\frac{FAR+FRR}{2})$$

where the false acceptance rate (FAR) estimates the risk that an unauthorized person would be mistakenly accepted by the system, and the false rejection rate (FRR) calculates the likelihood that a person with authorization will be mistakenly rejected by the system. A threshold value is used to determine the best values for FRR and FAR. Any change in the threshold’s value will directly affect FAR and FRR. To determine the verification accuracy, FRR and FAR are combined to their optimal value. When the verification accuracy is the highest, we have determined that the FRR and FAR combination is the best option for the suggested approach.

## 4. Results and Discussion

**Figure 4:**
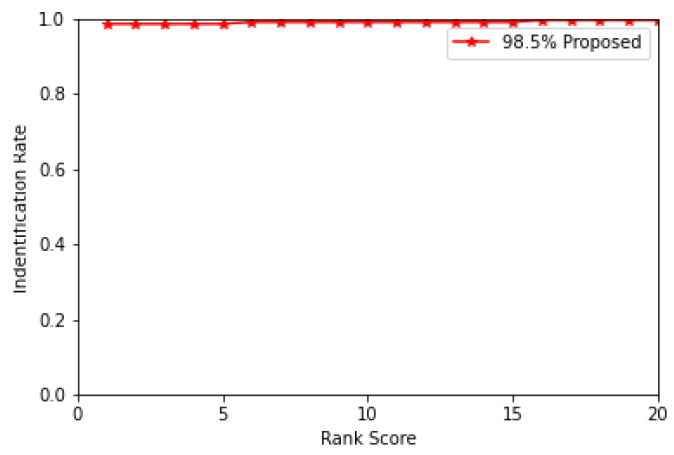
Identification rate on the UND J2 dataset.

The experiment on the UND J2 dataset shows 98.51% rank-1 accuracy. The cumulative match characteristic curve (CMC) for the top 20 ranks on the UND J2 dataset is illustrated in Figure 4.

The top-performing distance metric was SP distance. A comparison among different distance metrics is shown in Table 1. The reported results are based on one sample per person, where the probes were selected randomly during this experiment. The SP distance metrics utilize Spearman’s correlation (SC) to rank the observations. Generally, the SC is suitable when the continuous data do not follow a line and have a monotonic relationship or ordinal data. In the case of a monotonic relationship, when one variable increases, the other variable shows either an increase or a decrease. Furthermore, this relationship does not have to be necessarily in a straight line. This aspect of Spearman’s correlation allows fitting curvilinear relationships. As our extracted feature data points are continuous with a monotonic relationship and ranked using Spearman’s correlation, SP distance metrics exhibit better results than other metrics.

**Figure 5:**
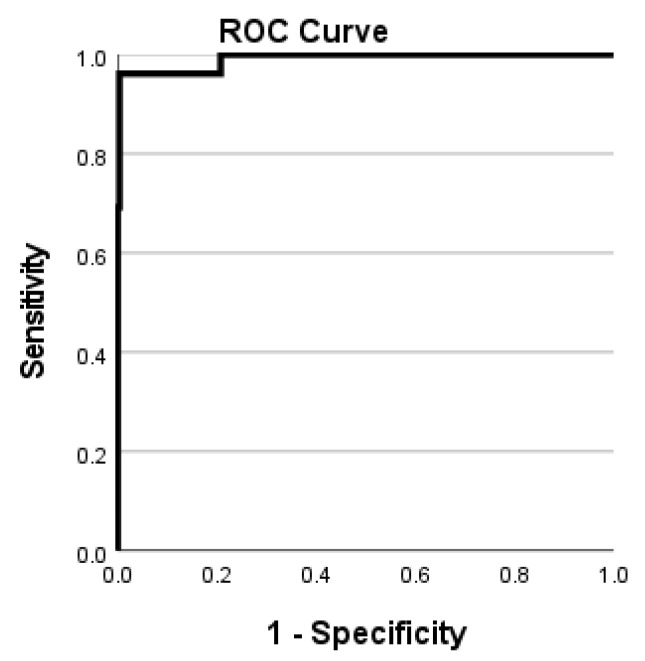
Performance evaluation of the proposed method on the UND J2 dataset using the ROC curve.

We empirically found the threshold value 0.62, which provides the best results to verify a person as authentic or an imposter. The verification accuracy was 97% (according to Equation (16)). We have also measured the verification performance in terms of the receiver operating characteristic curve (ROC), which is shown in Figure 5.

**Figure 6:**
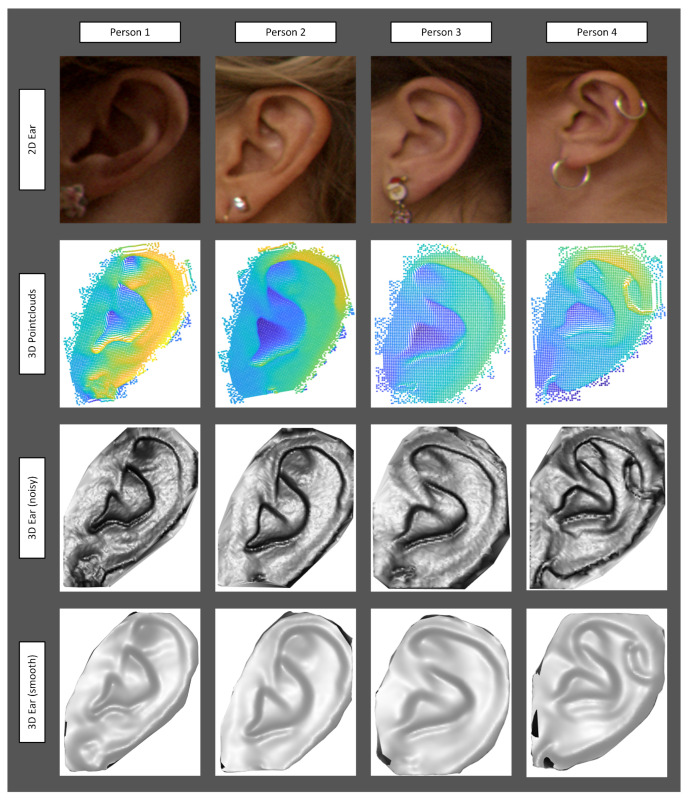
Examples of occlusions by earrings.

The proposed method performed well in the presence of natural hair near the ear region. Furthermore, we found that the proposed technique is able to recognize a person in the presence of earrings. Examples of ear recognition in the presence of earrings are illustrated in Figure 6. Additionally, we also demonstrate the performance by adding synthetic noise in the ear images. We found that the recognition performance reduces when adding more than 40% of noise.

Compared to the other recognition systems shown in Table 2, the ear recognition system from this article exhibits greater identification accuracy. All the other techniques used one-to-one matching using registration-based algorithms. As a result, the computational time increase for a large gallery dataset. On the other hand, our proposed YMO algorithm deforms the 3DMEM to a given probe for extracting the shape parameters. After obtaining the parameters, it does not require additional one-to-one registration between the gallery and the proof; rather, it only calculates the distance from the gallery of shape parameters. Therefore, the computation time is not increased even for larger gallery datasets.

As the algorithms were applied in various hardware settings, the time cost in the table is simply for reference. Nevertheless, it is possible to gauge these algorithms’ effectiveness using their computational complexity. Each ICP registration has a computational cost of $O(Iter*N_{p}log_{2}\left(N_{g}\right))$, where Iter is the number of iterations, $N_{p}$ is the number of probe data points, and $N_{g}$ is the number of gallery ear data points. The YMO algorithm converges to the minimum distance with fewer iterations when using the proposed recognition approach, which lowers the ear data to one-third the size of coarsely extracted ear area data. Therefore this ear recognition method is more efficient due to its reduced computational complexity. The average computation time to perform the recognition through a pairwise matching between probe and gallery templates for [26] was around 0.0039 s, while our method requires 0.0035 s. At the same time, [22] took 2.28 s for each probe and gallery pair, which is significantly large when the gallery data are increased. The computation time comparison is shown in Table 3.

## 5. Conclusions

In this work, a novel approach based on a 3D morphable model is proposed for human recognition using ear images. For identification, all gallery images were analyzed by the fitting algorithm named YMO, and the shape coefficients were stored. For a given probe image, the YMO algorithm calculated coefficients, which were then compared with all gallery data to find the nearest neighbor. The proposed method reduces the computational time by eliminating one-to-one registration between the probe and gallery, which only calculates the distance between the parameters stored in the gallery and the probe. The comparison among different state-of-the-art methods shows that our method can be implemented in a real-time scenario. The challenge of applying ear images for human recognition is acquiring ear images. Although the proposed method is robust against natural occlusions, for effective recognition, it is obvious to capture the ear image with minimal occlusions, such as hair, headphones, etc. In our future work, we will analyze the method in more datasets and deploy it in edge devices for biometric applications.

## Figures and Tables

**Table 1:** Accuracy comparison among different distance metrics.

Distance Metrics	Correct/Wrong	Rank-1 Accuracy (%)
CH	273/131	67.57
MN	347/57	85.89
SE	347/57	85.89
E	347/57	85.89
CR	351/53	86.88
MH	351/53	86.88
CS	352/52	87.13
CT	381/23	94.31
SP	398/6	98.51

**Table 2:** The performance comparison in terms of rank-1 recognition accuracy of the proposed technique with existing state-of-the-art techniques in the literature.

Authors	Recognition Approach	Identification Rate (%)
Islam et al. [22]	L3DF and ICP	93.50
Prakash et al. [23]	SURF and GPA(ICP)	98.30
Yan et al. [44]	ICP	97.80
Sun et al. [25]	Key-point matching	95.1
Chen et al. [15]	LSP and ICP	96.36
This work	3DMEM and YMO	98.51

**Table 3:** The comparison of computation time for the recognition phase of the proposed technique with existing state-of-the-art methods.

Authors	Mean Computation Time/Probe (s)
Islam et al. [22]	2.28
Jindan et al. [45]	0.019
Maity et al. [26]	0.0039
This work	0.0035 ± 0.0002

## Data Availability

Not applicable.
